# Fast Growing, Healthy and Resident Green Turtles (*Chelonia mydas*) at Two Neritic Sites in the Central and Northern Coast of Peru: Implications for Conservation

**DOI:** 10.1371/journal.pone.0113068

**Published:** 2014-11-19

**Authors:** Ximena Velez-Zuazo, Javier Quiñones, Aldo S. Pacheco, Luciana Klinge, Evelyn Paredes, Sixto Quispe, Shaleyla Kelez

**Affiliations:** 1 Department of Biology, University of Puerto Rico-Rio Piedras, San Juan, Puerto Rico; 2 ecOceánica, Lima, Perú; 3 Laboratorio Costero de Pisco, Instituto del Mar del Perú, Ica, Perú; 4 Instituto de Ciencias Naturales Alexander von Humboldt, CENSOR Laboratory, Universidad de Antofagasta, Antofagasta, Chile; 5 Unidad de Investigaciones en Depredadores Superiores, Instituto del Mar del Perú, Callao, Perú; UC Santa Cruz Department of Ecology and Evolutionary Biology, United States of America

## Abstract

In order to enhance protection and conservation strategies for endangered green turtles (*Chelonia mydas*), the identification of neritic habitats where this species aggregates is mandatory. Herein, we present new information about the population parameters and residence time of two neritic aggregations from 2010 to 2013; one in an upwelling dominated site (Paracas ∼14°S) and the other in an ecotone zone from upwelling to warm equatorial conditions (El Ñuro ∼4°S) in the Southeast Pacific. We predicted proportionally more adult individuals would occur in the ecotone site; whereas in the site dominated by an upwelling juvenile individuals would predominate. At El Ñuro, the population was composed by (15.3%) of juveniles, (74.9%) sub-adults, and (9.8%) adults, with an adult sex ratio of 1.16 males per female. Times of residence in the area ranged between a minimum of 121 and a maximum of 1015 days (mean 331.1 days). At Paracas the population was composed by (72%) of juveniles and (28%) sub-adults, no adults were recorded, thus supporting the development habitat hypothesis stating that throughout the neritic distribution there are sites exclusively occupied by juveniles. Residence time ranged between a minimum of 65 days and a maximum of 680 days (mean 236.1). High growth rates and body condition index values were estimated suggesting healthy individuals at both study sites. The population traits recorded at both sites suggested that conditions found in Peruvian neritic waters may contribute to the recovery of South Pacific green turtles. However, both aggregations are still at jeopardy due to pollution, bycatch and illegal catch and thus require immediate enforcing of conservation measurements.

## Introduction

Green turtles (*Chelonia mydas*) experience ontogenic habitat changes during their life cycle, switching from a juvenile oceanic epipelagic phase where they drift and disperse for several years [1]–[3], to a juvenile, sub-adult and adult neritic phase, where they feed and grow to reach sexual maturity [4], [5]. During the neritic phase, green turtles inhabit diverse ecosystems [5] such as sea grass beds [4], [6], mixed-bottoms composed of algae, sea grass and mangroves [7]–[12], and coral reefs, but usually nearby sea grass or algae beds for foraging [13]. All of these ecosystems are considered important development habitats of juveniles until reaching sexual maturity [3]. These areas can also be shared with adults who use them for foraging in between reproductive periods [5], [14]. Given the considerable time spent in these neritic areas, studies at these habitats represent unique opportunities to understand fundamental aspects of their life history, population dynamics [15]–[17], feeding ecology [18], [8], and functional role in the ecosystem [19]. Furthermore, the fact that green turtles are still endangered as a result of unsustainable practices (e.g., indiscriminate hunt for human consumption), habitat degradation and fisheries by-catch [20], [21] studies in neritic habitats provide highly valuable information to propose conservation actions for this species such as preserving the habitat quality and the resources they feed on [22].

The Southeast Pacific holds a large aggregation of green turtles [23] recruiting from different nesting areas around the tropical-equatorial realm. In this region, this species may encounter different neritic conditions such as those imposed by the cold, nutrient-rich, upwelling coastal waters of the Humboldt Current System extending from central Chile (∼35°S) to northern Peru (∼6°S), and a transitional area (∼6°–3°S) characterized by the convergence of northward upwelling waters with the warm, less-productive waters coming from the equator and warm tropical waters that predominate in the coastal area of the northern tip of Peru towards Ecuador [24]. These conditions influence the existence and boundaries of different ecoregions [25] where green turtles may find a variety of foraging resources from distinct habitats ranging from coastal upwelling embayments to mangrove areas. Green turtles also have nesting rookeries located within the tropical realm [26], [27].

Overall, the functionality of the different neritic habitats and their relationship with the distribution of each green turtle life stage within this region is still poorly understood and the information available is far from conclusive. A single study, based on the analyses of routes taken by post-nesting females from Galapagos, suggests sea surface temperature (SST) ≤25°C may act as a barrier to migration [28], but this might only be affecting adult females since juvenile and sub-adult green turtles are present in areas with lower SST. In central-south Peru, green turtles are abundant in warm neritic waters occurring during El Niño events [29]. Indeed, ca. 1000 tons of green turtles were captured and landed by artisanal fishermen during the El Niño 1987 [30]. However, landing data combines all turtle catch sites, thus precluding the assessment of habitat-life stage relationships. Green turtles can also be present in colder and temperate neritic habitats [8]. In Peru, green turtle aggregations have been registered inhabiting several areas along the coastline like Paracas bay ∼14°S [29], [30], [31], Tambo de Mora ∼13.3°S, Lobos de Afuera island ∼7°S, Lobos de Tierra island ∼6.5°S, Sechura bay ∼5.7°S, Casitas y Punta Restin ∼4.5°S and several areas along the coast of Tumbes (Punta Sal, Punta Mero, Bocapan, Puerto Pizarro) ∼3.5–4°S [29], [31], [32]. Yet, the roles of these different habitats in the population structure need to be evaluated.

Neritic habitats off Peru might constitute important feeding grounds for this species. Results from gut content studies suggest remarkable differences in the diet composition of green turtles from northern [33] and south-central sites along the coast of Peru [29], [30], [34]. These differences in food resources could reflect differences in the composition of the aggregation; thus, north versus south differences in life-stage distribution could also be expected. Furthermore, given that breeding areas are located in the equatorial realm, dispersal of post-hatchling individuals occurs towards southern locations of Peru thus southern neritic benthic habitats could be dominated by recruiting juveniles (i.e., developmental habitats) [16], while a mixed group composed by juveniles, sub-adults and adults could be most common in neritic habitats at the most northern locations in the coast of Peru.

Here we provide new information of two aggregations in an upwelling and an ecotone (Humboldt-Equatorial) site. The aims of this study were: (1) to estimate and compare population parameters at each site such as size frequencies, growth rates, and sex ratios in adults, and (2) to estimate and compare time of residency at each site. We predict that there will be proportionally more adult individuals in the ecotone site compared to the upwelling site where juvenile individuals would be relatively more abundant.

## Materials and Methods

### Ethics statements

According to the Ley Forestal y de Fauna Silvestre del Perú N° 29763 (Forestry and Wildlife Law of Peru) and its Reglamento de la Ley Forestal y de Fauna Silvestre through a Decreto Supremo DS No 014-2001-AG (Supreme Decree for regulation) requires permissions for conducting any research activity involving CITES (Convention of International Trade in Endangered Species of Wild Fauna and Flora) enlisted species present in the Peruvian territory outside of national protected natural areas. The Servicio Nacional Forestal y de Fauna Silvestre (National Forest and Wildlife Service) grants permission upon request and after a technical opinion from a specialist indicating that the research activities will not jeopardize the conservation status of endangered species. All research activities at El Ñuro were conducted under permits RD N° 0383-2010-AG-DGFFS-DGEFFS and RD N°0606-2011-AG-DGFFS-DGEFFS. The Servicio Nacional de Areas Naturales Protegidas (National Service for Natural Protected Areas) provided the permissions for conducting sea turtle research in Paracas National Reserve (N208-2013-SERNANP-RNP/J, N105-2013- SERNANP-RNP/J, N087-2011- SERNANP-RNP/J). All permits allowed the manipulation and tagging of the animals as well as the collection of small tissue samples. All turtles were handled by trained personnel and following research and ethical protocols as in other sea turtle projects available in [35]. To minimize distress on captured turtles we kept them aboard for a maximum of 20 minutes, each individual was covered with wet towels to reduce overheating, and we released them in the same location were originally captured. In this study, no sea turtles were injured or killed during sampling manipulations.

### Study locations

#### El Ñuro - the ecotone site

**Figure 1:**
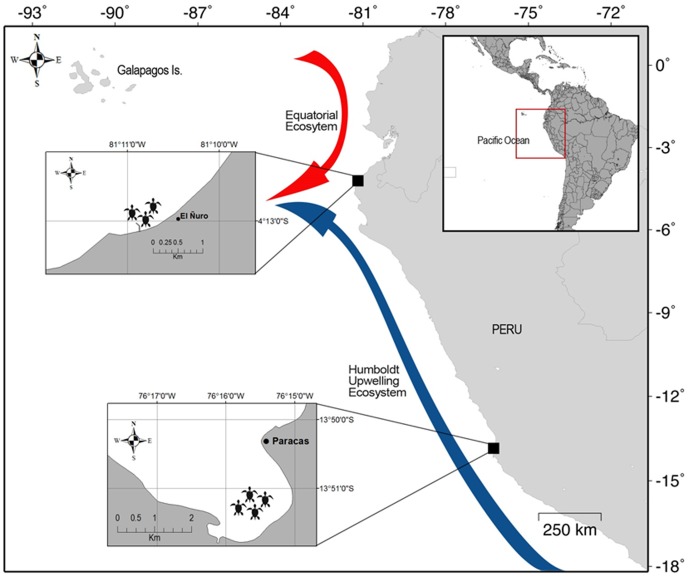
Green turtle (*Chelonia mydas*) neritic aggregations at two sites along the coast of Peru. (A) El Ñuro, at the northern part of the Peruvian coast, a region characterized by the convergence of the Humboldt upwelling ecosystem and the warm Equatorial ecosystem and (B) Paracas, characterized by permanent upwelling in the central coast of Peru. Map was created using Maptool available at www.seaturtle.org/maptool/.

In-water surveys for green turtles were conducted in El Ñuro (4°13′S; 81°10′W, Fig. 1), a large sandy neritic area in the coast of the department of Piura, northern Peru. Green turtles were sampled in an area to the north-east of a pier in this area. As mentioned before, this area represents an ecotone between the cold upwelling waters of the Warm Temperate Southeastern Pacific Ecoregion and the warm and tropical conditions of the Tropical Eastern Pacific Ecoregion [25]. Sea surface temperature (SST) during the study period was on average 22.2°C (range: 19.3°–25.9°C). The seabed at this area is mainly composed by sandy sediment with scattered boulder reefs. The rocks and to some extent, the sediment substratum, are covered by the invasive green algae *Caulerpa* sp. (authors observations). The El Ñuro pier is used by ca. 350 artisanal fishermen mainly targeting demersal Peruvian hake (*Merluccius gayi*), large oceanic fish such as tuna (*Thunnus albacares*) and swordfish (*Xiphias glaudius*), and Humboldt squid (*Dosidiscus gigas*). Fishermen conduct day-fishing trips leaving between 3 and 4 am and returning from 11 am to 4 pm.

A nylon entanglement net (80 m×5 m, mesh size = 20.32 cm) was used to capture green turtles. Each in-water survey lasted 5 days, each day the net was deployed for around 5 hours usually starting at 8am and finishing at 1pm. The bottom part of the net was generally on top of the sea floor at ∼5 m depth. In order to attract sea turtles to the net, some small pieces of Humboldt squid mantle were tied to the upper part of the net. Two to four free divers monitored the net every 30 minutes searching for sea turtles and helping to bring them on-board. A total of eight in-water surveys were conducted from the 31^st^ of May 2010 to the 16^th^ of August 2013, every 23.3 weeks on average (SD ±5.3).

#### Paracas – the upwelling site

In-water surveys were conducted at La Aguada a small inlet located in the South Eastern part of Bahia Paracas, in central Peru (13°51′S; 76°15′W, Fig. 1). These shallow waters are located within a cold temperate upwelling region, being one of the main feeding grounds in the Southeast Pacific [36]. SST during the study averaged 18.8°C (range: 15.1°–22.8°C). Green turtles were sampled within an estimated area of *ca.* 3.9 km^2^. The seabed in this area is mostly sand covered by algae *Ulva* sp., *Enteromorpha* sp., *Chondracanthus* sp. [37] and *Caulerpa* sp. During the austral spring, summer and autumn, the pelagic medusae phase of the large jellyfish *Chrysaora plocamia* is very abundant [38]. This area is used by few fishermen targeting fish species such as mullets (*Mugil cephalus*) and silversides (*Odontesthes regia regia*).

A set of four entanglement nets (total length: 976 m×4 m, mesh size = 60–65 cm), traditionally used by local artisanal fisherman for catching sea turtles, was used. Each in-water survey lasted 2 days and the nets were deployed randomly within the area, usually starting at 5am and finishing at 1pm, around 8 hours on average. The net operates just below of the water column surface with no bait, as traditionally used by local artisanal fishermen in this area. When a sea turtle was captured we approached the net sailing and carefully brought it on board for evaluation. A total of 21 in-water surveys were conducted every 8 weeks on average (SD±7.9) from the 4^th^ of March 2010 to the 12^th^ June 2013.

### Collection of biological data at both sites

At both sites the following body measurements were taken from each turtle captured: the notch to tip Curved Carapace Length and Curved Carapace Width (CCLn-t, CCWn-t) and two measurements of the tail (Total Tail Length-TTL and Post-cloacal Tail Length) to 0.1 cm with a 100 cm soft measuring tape. In El Ñuro, notch to tip Straight Carapace Length and Straight Carapace Width (SCLn-t, SCWn-t) to 0.1 cm was obtained using a 100 cm Haglöf tree caliper. In Paracas, SCLn-t was not measured but estimated using the regression formula SCLn-t = 0.919CCLn-t+1.9645 (R^2^ = 0.9845) estimated from turtles at El Ñuro. Green turtles were weighted using a 100 or 200 Kg scale (1 kg precision). For the largest turtles at El Ñuro, weight was not obtained using a scale, but estimated using the regression equation between size (CCLn-t) and weight (W): W = 1.916 (CCLn-t)–87.611, R^2^ = 0.91; estimated using data of smaller individuals. In Paracas all turtles were weighed. The sex of the turtles was determined based on the secondary sexual characteristics i.e. length of the tail and nails. All captured green turtles were marked with external Inconel tags. At El Ñuro, tags were always applied in both front flippers, except for nine individuals were tags were applied in only one of their flippers. In Paracas, Inconel tags were applied at both back flippers.

### Population parameters, residency time, growth rate and body condition

Green turtles were arbitrarily classified in three different life-stages (i.e., juvenile, sub-adult and adult) using the minimum and mean CCLn-t size of nesting females in Galapagos (Patricia Zarate, personal communication). All individuals with CCLn-t, at first capture, under the minimum size of nesting females (<60.7 cm) were categorized as juveniles, all between the minimum and the mean size (≥60.7 and ≤86.7 cm) as sub-adults, and all over the mean (>86.7 cm) as adults. Residence time was estimated as the time period elapsed between the first capture of the turtle and the last recapture and was expressed in days. Growth rates were estimated from the capture-recapture records as the increments in size (both for CCLn-t and SCLn-t) in cm per year. The body condition index (BCI) was calculated using the following formula: BCI = body mass/SCL [3]. This index is used as an indirect predictor of the nutritional status and/or health condition of the animal [39]. To calculate growth rates and BCI, only recapture intervals greater than 300 days were used, therefore reducing the effect of measurement error and seasonality effects [40], [41], [42].

### Statistical analysis

Green turtle size (CCLn-t and SCLn-t) and weight were compared using a two- way PERMANOVA test considering site (Ñuro and Paracas) and sampling years (2010–2013) as fixed factors. PERMANOVA is a semi-parametric test based on Euclidian distance and calculates the significance using permutations. This test is robust enough in cases when data normality and variance homogeneity is not achieved such as in the case of our data set. This analysis also allows testing for significant effects of the interaction term (i.e. site×sampling years). Body condition index values were compared using PERMANCOVA test using a three-way design, considering CCLn-t as a co-variable and life stage (juveniles and subadults), site (Ñuro and Paracas), and years (2010–2013). Since adults were present only in el Ñuro, body condition index values of this life stage were not included in the statistical test. All statistical tests were performed using the PRIMER v6+PERMANOVA software [43].

## Results

### Number of capture and recapture individuals

At el Ñuro, a total of 228 green turtles were captured, with an average of 28.4 individuals per survey (range: 5–45). Of the total captured individuals, 154 were unique individuals while 51 were recaptures among surveys, 20 within surveys, and three captures from which data was not possible to get collected but it was used for CPUE estimates. For all subsequent analyses, within-survey recaptures were excluded. The mean number of new captures and recaptures was 19.5 new turtles per survey (range: 4–31, SD ±10.4) and 6.4 turtles (range: 0–13, SD ±4.5) respectively. One turtle, an adult female, had a tag from the Galapagos archipelago, applied in a nesting beach at Bahia Barahona, Isla Isabela, in 2010 (Macarena Parra personal communication). Also, two juvenile hawksbills (*Eretmochelys inmbricata*) were captured during the surveys. Two tagged green turtle from our project were found dead, one at El Ñuro and one at Los Organos, a beach 8 km to the north of El Ñuro. At Paracas, a total of 160 green turtles were captured, with an average of 5.3 individuals per survey (range: 1–27). Of the total captured turtles, 133 were unique individuals while 27 were recaptures among surveys. All turtles captured within surveys were excluded. The mean number of new captures and recaptures was 7.2 (range: 1–27, SD ±5.7) and 0.7 turtles (range: 0–5, SD ±1.3) respectively. One adult olive ridley sea turtle (*Lepidochelys olivacea*) was captured during surveys. In addition, one tagged turtle was found dead in the Pisco shores.

### Size, weight, size classes and sex ratio

**Figure 2:**
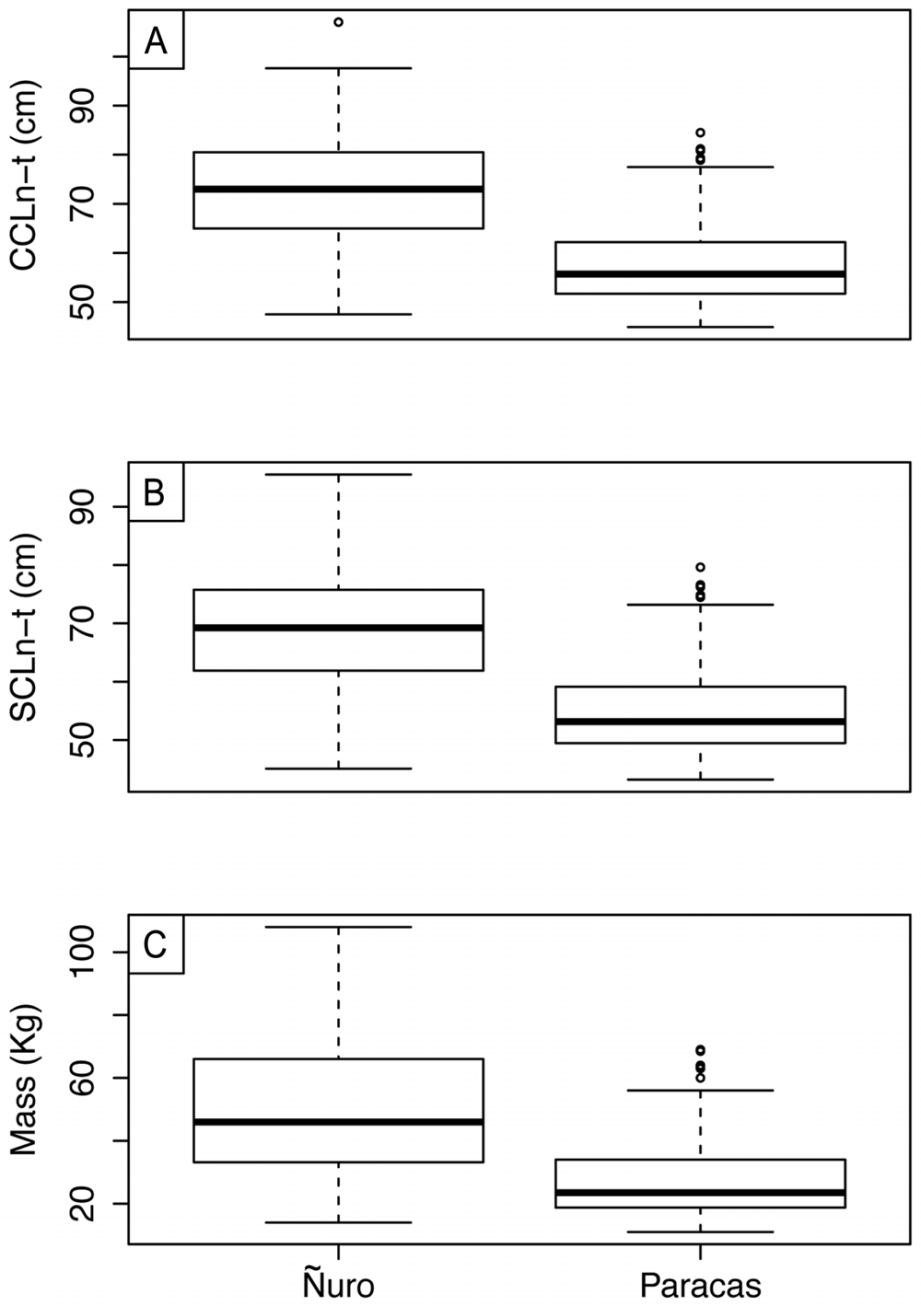
Box plots of (A) curve carapace length, (B) straight carapace length and (C) body weight of green turtles at both study sites.

Overall, green turtles at El Ñuro were larger and heavier compared to the individuals captured at Paracas (Fig. 2, detailed body measurements are provided in Table S1). The size (CCLn-t) of captured green turtles at el Ñuro varied between 47.5 cm and 107 cm, with a mean size of 72.4 cm (SD ±10.9), while at Paracas the size ranged from 44.9 to 84.5 cm, with an average size of 57.7 cm (SD ±8.7). The PERMANOVA test indicated that differences for both CCLn-t (Pseudo-F_1, 356_ = 196.9, P<0.05) and SCLn-t (Pseudo-F_1, 356_ = 192.9, P<0.05) were significant. In addition, the interaction term time×year was also significant (CCLn-t; Pseudo-F_3, 356_ = 3.1, P<0.05 and SCLn-t; Pseudo-F_3, 356_ = 2.9, P<0.05) indicating that significant differences were persisting during all sampling years. At El Ñuro, green turtles weighed on average 50.2 kg (SD ±21.4), ranging from 14 to 108 kg while at Paracas individuals weighted on average 27.7 kg (range: 11–69 kg, n = 160, SD ±13.0). The PERMANOVA test also detected significant differences in weight (Pseudo-F_1, 356_ = 146.2, P<0.05) and the interaction term time×year (Pseudo-F_3, 356_ = 3.3, P<0.05).

**Figure 3:**
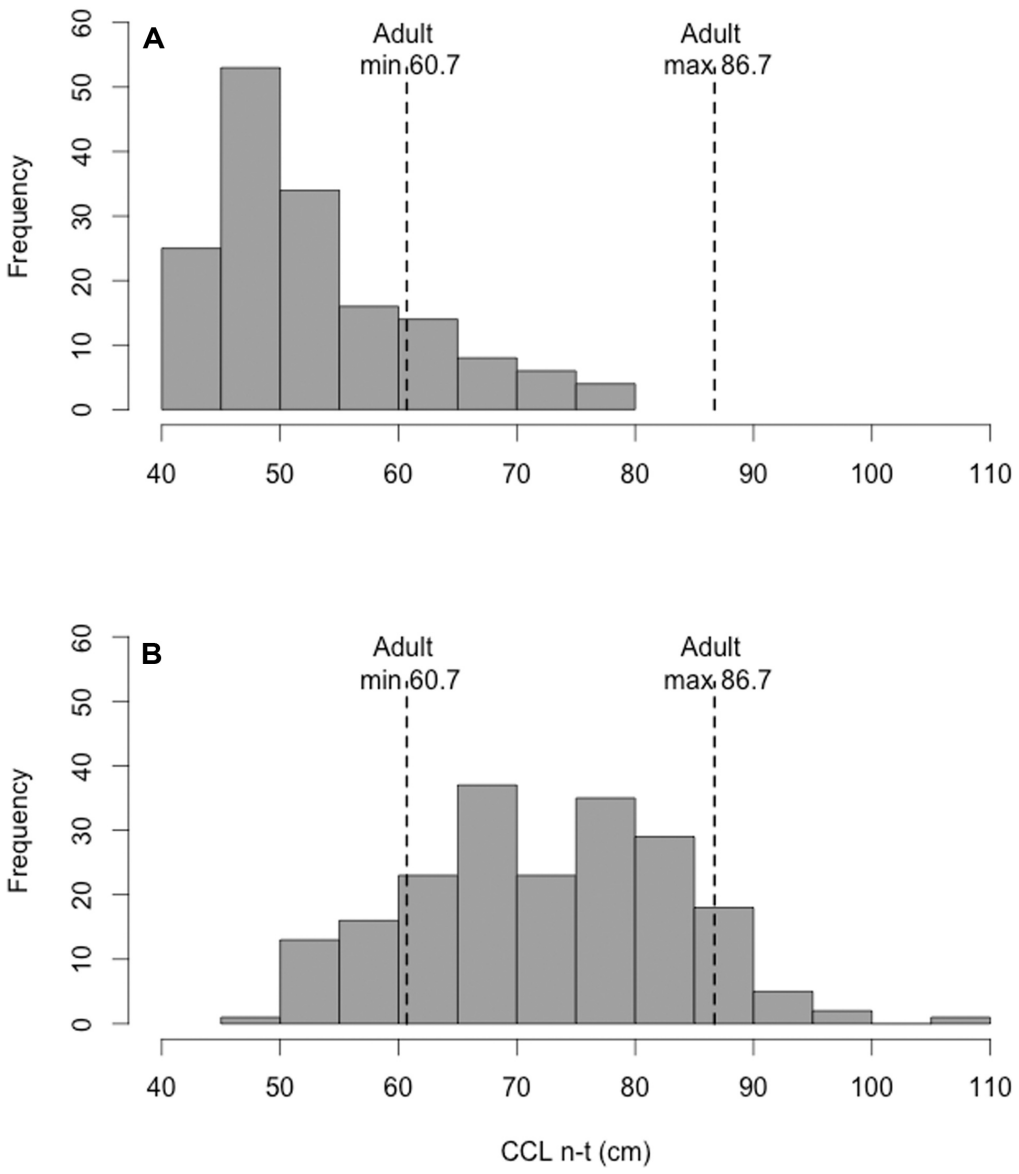
Size (CCLn-t) distribution of green turtles captured at (A) Paracas and (B) el Ñuro. Dotted lines are the minimum and mean size reported for adult green turtles in Galapagos Island [36], the largest green turtle rookery near Peru.

At El Ñuro, the majority of individuals (81.7%; n = 166) were between 70 and 89 cm length (Figure 3) and 74.9% (n = 152) of the captured individuals were classified as sub-adults, 15.3% (n = 31) as juveniles and 9.9% (n = 20) as adults (Figure 3). The mean CCLn-t of adult individuals was 90.6 cm (range = 86.7–107, SD ±4.9). At Paracas, 73% (n = 117) individuals were classified as juveniles and 27% (n = 43) sub-adults; no adults were captured (Fig. 3). The mean CCLn-t for juveniles was 53.1 (range: 44.9–60.4 cm, SD ±3.7) and for sub-adults 69.5 (range: 61.3–84.5 cm, SD ±6.5). At El Ñuro, from all unique turtles captured, eight were identified as adult females and seven were identified as adult males. The mean TTL for adult males, including measurements obtained in subsequent recaptures, was 30.9 cm (range = 19–45 cm, SD ±7.8). These males ranged in size (CCLn-t) from 71.6 to 83.5 cm and averaged 79.4 cm. The sex ratio in captured adult sea turtles was 0.85 F∶ 1M. Since at Paracas no adults were recorded, the proportion of males and females of this aggregation remain unknown.

### Time of residency, growth rates and body condition index

**Figure 4:**
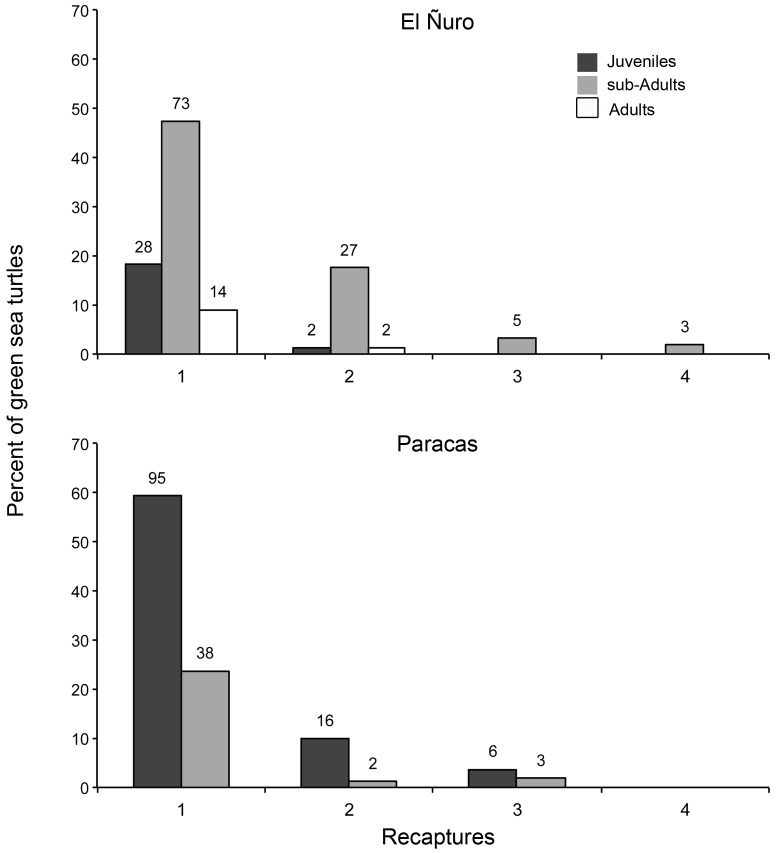
Number of times (%) that each turtle classified as juveniles (dark gray bar), sub-adult (light gray bar), or adult (white bar) was recaptured in El Ñuro and Paracas. The total number of turtles caught is indicated on top of each column.

At El Ñuro, the mean recapture rate was 26%. In general, most of the individuals evaluated were captured only once (n = 115) but there were turtles captured more than once during these four years of surveys (two times: n = 31; three times: n = 15; four times: n = 3). There were more sub-adult turtles recaptured in comparison to the other life-stages (Fig. 4). The mean recapture time interval was 331.1 days (SD ±215.4), with a minimum of 121 days and a maximum recapture interval of 1015 days. At Paracas, the mean recapture rate was 12.5%. Most of the individuals evaluated were captured only once (n = 133), a few twice (n = 18) and only nine turtles were captured three times during these four years of surveys. There were more juvenile turtles recaptured in comparison to sub-adults (Fig. 4). The mean recapture interval was 277 days (SD ±236.2), with a minimum of 65 days and a maximum recapture interval of 680 days.

**Figure 5:**
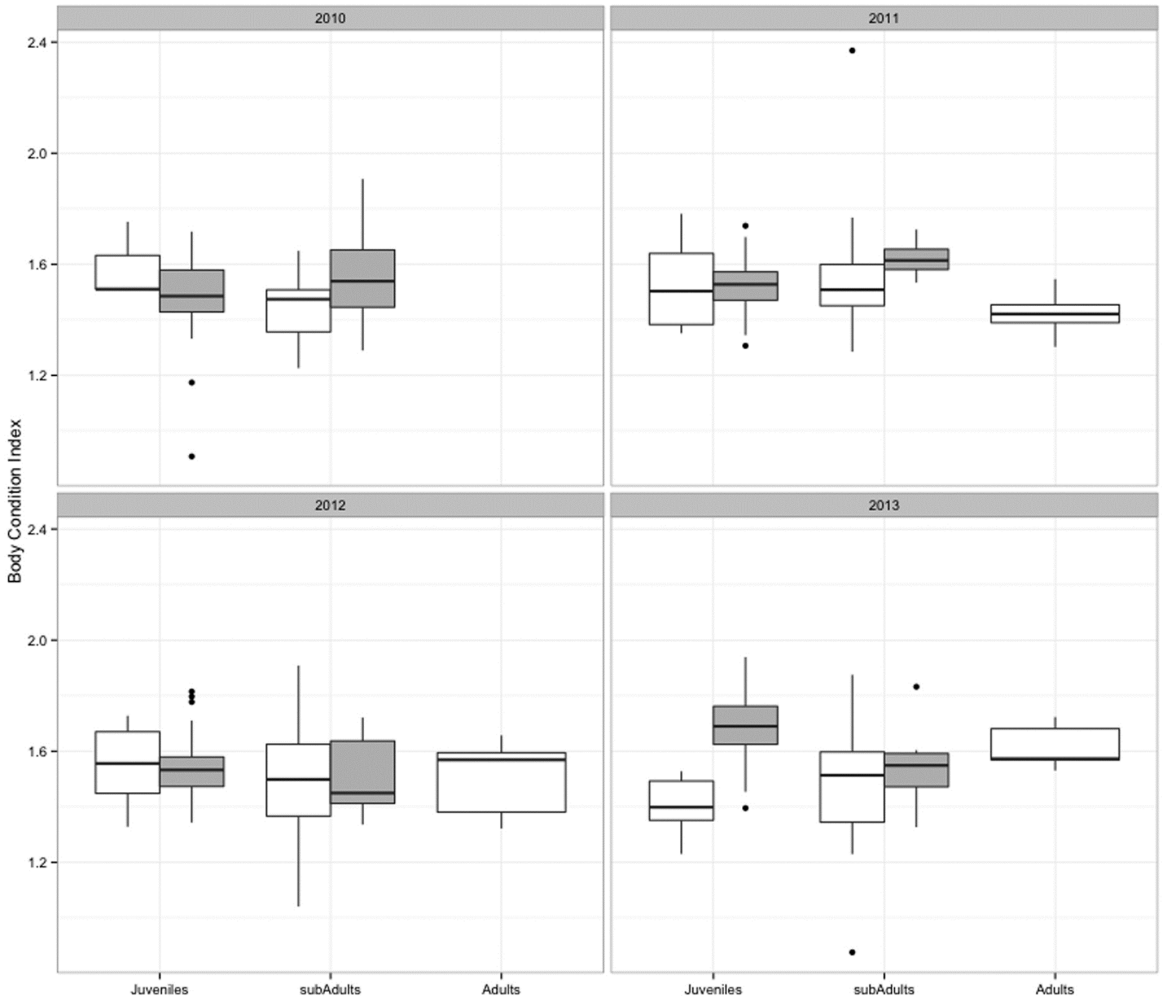
Box plots of body conditions values per green turtle life stages at the different sampling years at El Ñuro (grey boxes) and Paracas (white boxes).

At El Ñuro, from all 45 recapture records (sub-adult = 44, adult = 1), we used 20 records with recapture intervals equal or higher than 300 days. The mean growth rate was estimated at 2.83±1.51 cm year^−1^ (range: −0.1–5.58). At Paracas, three usable recaptures yielded a mean growth rate of 6.77±1.75 cm year^−1^ (range: 4.77–8.07). Since we did not have equally and sufficient recaptures of individuals in all life stages, particularly from Paracas, no contrasting statistics were performed. Body condition index values at El Ñuro showed slight variation among life-stages and years. The lowest BCI exhibited per year and stage was for the juveniles in 2013 (BCI = 1.4) while adult green turtles exhibited the highest BCI in 2013 (1.6). In general, the mean BCI for each life stage (juveniles, sub-adults and adults) was 1.5. At Paracas, estimates of BCI were quite similar among life-stages and years (Fig. 5). Juvenile green turtles exhibited both, the lowest and maximum BCI (2010, BCI = 1.4 and 2013, BCI = 1.6 respectively) while adult green turtles exhibited similar values; the mean BCI for juveniles and sub-adults was the same (1.5). The PERMANCOVA test comparing BCI values of juveniles and sub-adults (excluding adults since these where only present at El Ñuro) detected marginal differences for the interaction term co-variable (CCL)×year×site (Pseudo-F_3, 281_ = 2.6814, P = 0.053).

## Discussion

This is the first study comparing two neritic aggregations of green turtles in the Southeast Pacific. The presence of almost exclusively juvenile individuals in the upwelling site (La Aguada in Paracas) provides support to the development habitat hypothesis supporting the role of neritic habitats exclusively used by juveniles throughout the benthic life stage. Overall, the results of our study highlight the importance of neritic habitats for juveniles, sub-adults and adults green turtles in Peruvian waters and in the eastern Pacific in general. Preliminary growth rates and body index condition estimates suggest that green turtles at both sites are thriving in very favorable habitats exhibiting the highest values reported for foraging populations in the eastern Pacific.

The distribution of green turtles life-stages along these two sites suggests a spatial segregation pattern between sites; while the El Ñuro aggregation is composed of mainly sub-adults, with few juveniles and adults, for green turtles caught in Paracas the aggregation is dominated by juveniles and few sub-adults. Paracas is located within one of the most productive and permanent upwelling cells along the Peru-Chile coast [44]. In this area, early studies on the diet of this species suggested macroalgae as their principal prey item [29]. Recently, green turtles in this area have been reported preying intensively on the large jellyfish *Chrysaora plocamia* (up to 100 cm in bell diameter) [30], [45], [46] that can be very abundant in the water column, particularly during summer [38]. Upwelling is less intense and more seasonal in northern Peru, but still highly productive conditions exist there. At El Ñuro, the diversity and abundance of seaweeds is low; however, the algae *Caulerpa* sp. is very common and distributed in large patches along the seabed. Green turtles have been observed having pieces of this algae sticking out of their mouths (authors observations). However, we do not know whether green turtles really feed on this algae or this is unintentionally trapped in the mouth when disturbing the seabed searching for other benthic prey. Also, we cannot rule out the possibility of the influence of an external source of food (e.g., fish discard), particularly near El Ñuro pier. Diet studies complemented with isotope data are necessary to understand the trophic ecology at El Ñuro.

**Figure 6:**
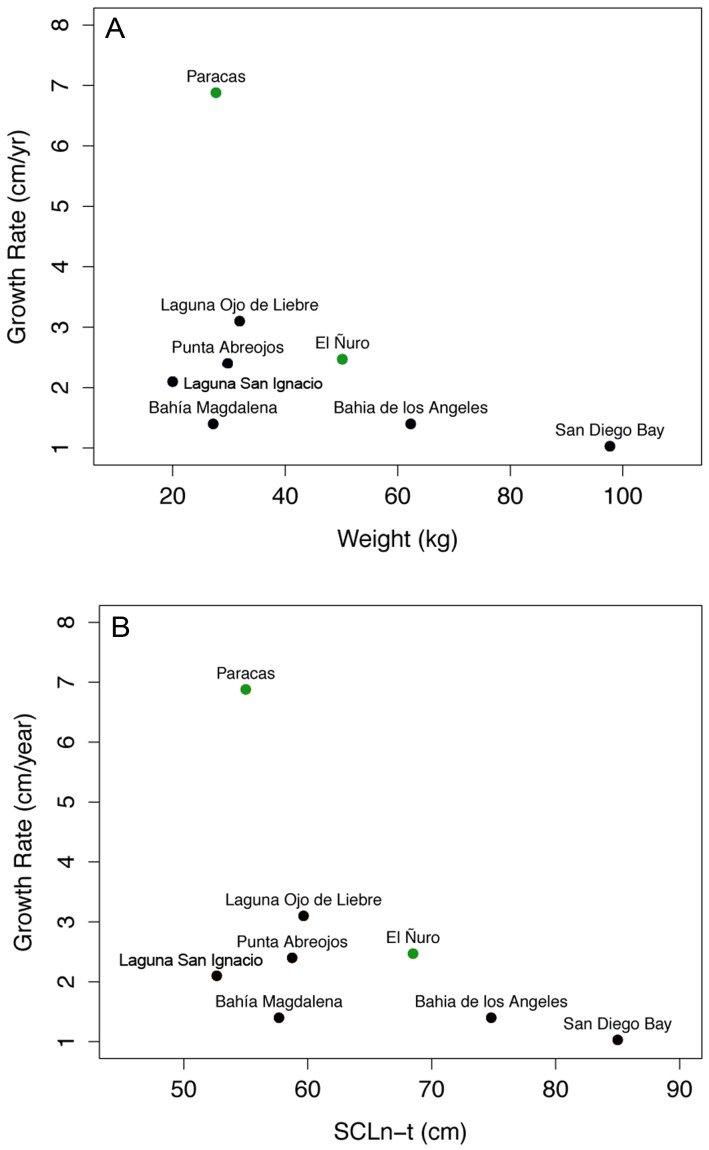
Relationship between weight and growth rate (A) and length (SCLn-t) and growth rate (B) for foraging aggregations of green sea turtles in the eastern and central Pacific (black dots), including our study (green dots).

Our estimates of growth rates suggest that the productivity may be playing a major role in the development of juveniles and sub-adults. Growth rate estimates obtained from green turtles at El Ñuro were the second highest estimates reported so far, after estimates obtained for the green turtle aggregation in Laguna Ojo de Liebre, in Mexico [47]. The green turtle aggregation from Paracas exhibited a high growth rate as well (6.8 cm/y ±1.8), but this estimate is preliminary and should be taken with caution. The growth rate estimates for Paracas were obtained from three recapture records, after filtering the dataset to comply with the minimum recapture interval established. Yet, these first growth rate estimates, at both sites, were high and individuals, particularly at Paracas, may be growing faster than individuals at El Ñuro. This difference is somehow expected since individuals at Paracas are significantly smaller individuals (mean CCLn-t = 57.7 cm) compared to green turtles at El Ñuro (mean CCLn-t = 72.42 cm) and it is known that green turtles grow faster at smaller sizes e.g. [48], [49], [50]. Our results suggest that green turtles in Peru are growing faster than individuals at different locations in the central and northeast Pacific and this pattern is observed when comparing both weight and SCL versus growth rate (Fig. 6, Table 1). Even though the coastal region of Peru is located within tropical latitudes, the characteristics of the prevalent upwelling coastal system i.e. cold, low oxygen and nutrient-rich waters, resemble a more temperate realm extending northward close to the equator (∼4°S). Green turtles inhabiting temperate feeding grounds may boost their metabolism for growing faster compared to their counterparts living in warm habitats [51]. The combination of younger stages, low temperatures and high prey availability may yield fast growing green turtles.

**Table 1:** Size, preliminary growth rates, and body condition index of green turtles (*Chelonia mydas*) at foraging grounds in the eastern Pacific.

Study site	N	Juv∶Adt (% or ind)	CCL mean (±SD)	SCL mean (±SD)	Weight mean (±SD)	Growth rate mean (±SD)	BCI mean (±SD)	Habitat	Adult criteria	Reference
Palmyra Atoll	211	80∶20	69.7 (16.1)	----	44.6 (29.7)	----	----	Reef with algae cover	CCL>85 cm (Gulf of Carpentaria) [57]	[58]
San Diego Bay	210	----	----	85 (17.3)	97.7 (52.1)	1.03	----	Power plant	----	[59], [49]
Bahia de los Angeles	200	112∶88 ind	80.9	74.8 (SE 0.7)	62.3	1.4 (0.93)	1.42 (0.015)	Benthic community dominated by macroalgae	MNS at Michoacan (SCL>77,3 cm) [60], [61]	[8], [51]
Laguna Ojo de Liebre	137	96∶4	----	59.66 (10.39)	31.9 (18)	3.1 (2.2)	1.32 (0.16)	(Lagoon) *Zostera marina*, benthic macroalgae	MNS at Michoacan (SCL>77,3 cm)	[47]
Laguna San Ignacio	220	99∶1	----	52.66 (7.71)	20 (10.9)	2.1 cm (1.3)	1.27 (0.23)	(Lagoon) seagrass beds and mangrove swamps	MNS at Michoacan (SCL>77,3 cm)	[47]
Punta Abreojos	604	94∶6	----	58.74 (10.49)	29.8 (17.9)	2.4 (1.2)	1.34 (0.17)	(Lagoon) Mangrove forest, seagrass and algae	MNS at Michoacan (SCL>77,3 cm)	[47]
El Pardito	59	85∶15	----	65.95 (11.10)	41.8 (22.4)	----	1.38 (0.13)	Islet within Gulf of California	MNS at Michoacan (SCL>77,3 cm)	[47]
Bahía Magdalena	169	97∶3	----	57.68 (8.83)	27.2 (12.9)	1.4 (0.7)	1.32 (0.15)	(Lagoon) seagrass beds and mangrove	MNS at Michoacan (SCL>77,3 cm)	[47]
Gorgona National Park	86	83∶3 ind	----	58.4 (7.8)	28.8 (10.7)	----	----	Soft and sandy bottoms, coral and soft corals areas	ND	[62]
Isla Plata	68	----	61.56 (5.69)	----	27 (7.58)	----	----	----	----	[63]
El Ñuro	203	90∶10	72.4 (10.9)	68.5 (10.1)	50.2 (21.4)	2.8 (1.5)	1.5 (0.16)	Sandy bottom with patches of algae	MNS at Galapagos Island [3]	This study
Paracas	160	100∶0	57.7 (8.7)	55 (8.0)	27.7 (13)	6.8 (1.8)	1.55 (0.14)	Sandy bottom with patches of algae	MNS at Galapagos Island	This study

Information includes site name, samples size (n), proportion of juveniles to adults (Juv∶ Adt) reported either as a percentage or as total count of samples individuals, mean and standard deviation (SD) of curve carapace length (CCL), straight carapace length (SCL) in cm, weight in kg, growth rate in cm year^−1^, and body condition index (BCI). We include information of the habitat and the size criteria to distinguish juveniles from adults (mean nesting size-MNS of females).

In line with these results, BCI estimations for green sea turtles at both sites were high in comparison to other populations in the east Pacific. For example, in Mexico, mean estimates for different sites ranged from 1.27 to 1.42 while in Peru the mean estimates were 1.49 and 1.55. These suggest that green turtles from El Ñuro and Paracas are thriving in neritic waters of central and northern Peru and may have found excellent habitat conditions for fast growth and development. Juveniles and sub-adult individuals may take advantage of such conditions before migrating as adults into relatively less productive waters off the nesting areas in the equatorial region.

Coastal and oceanic waters off Peru have been described as “a major sink for marine turtles in the Pacific” due to the high number of bycatch of all sea turtles species [23]. The highly mobile and migratory nature of sea turtles and the fact that the Peruvian coast may constitute an important migratory-displacement habitat could explain the occurrence of such large by-catch. However, our study emphasizes the fact that green turtles use coastal waters in this region to spend considerable amounts of time at localized sites in the neritic realm. At El Ñuro individuals monitored from 2010 to 2013 have stayed or departed and returned at intervals of up to 1015 days (2.7 years) while at Paracas the maximum residency time observed is 680 days. Green turtles may stay at the same area because the habitat may provide enough food resources and refuge. As stated before, prey availability is high, particularly in Paracas, while in the seabed at El Ñuro turtles use several caves and crevices within rocky reefs (Aldo S. Pacheco personal observations) for resting which may facilitate the localized residence at this site. Recapture rates were higher at El Ñuro in comparison to Paracas; however, strict comparisons between sites should be taken with caution since capture strategies and efforts were different. Sampling at Paracas was done using considerable larger nets compared to the one deployed at El Ñuro. Despite these differences, some plausible explanations can be drawn. Low recapture rates at Paracas may imply that the population is rather large and individuals are constantly moving throughout the area, likely exploiting actively natural resources as in the likely case of juveniles recruiting from the oceanic habitat [3], [5]. In addition, we cannot rule out the possibility that the local conditions such as shallow depth of our sampling site is preferred by juveniles and that the adults may be distributed in deeper waters within the general area. The presence of almost exclusively juveniles provides support to the development habitat hypothesis, stating that habitats occupied only by juveniles may be found during the neritic stage as stated by Meylan et al. [16] who examined an extensive data set supporting this hypothesis for Caribbean green turtles. As an example of contradictory evidence they cite the work of Hays-Brown & Brown [29] reporting the presence of a mix of adult and juveniles individuals in Pisco, a few kilometers north of Paracas. However, Hays-Brown & Brown [29] present percentages of straight carapace length based on 416 individuals landed in the port of Pisco, but no indication is given regarding the localities where green turtles were caught. This precludes any assessment of size stage segregation between habitats in this whole area. Our data from La Aguada (Paracas) suggest that only juveniles may occur in specific sites. Nevertheless, the lack of adults can be also attributable to the effect of the former high fishery in Pisco-Paracas and the current illegal catch that may have depleted the number of adults in the area. Moreover, from 1976 larger animals were targeted by the legal fishery as a consequence of a ministerial decree which banned the capture of green turtles smaller than 80 cm of “total” length (not carapace length) until the complete closure of the fishery in 1995 [32]. In contrast, at El Ñuro sub-adults and adults were more common residents, possibly with less displacement movements given the differences of both areas. These turtles may use the area as a permanent residence habitat taking advantage of an additional food supply such as fish and squid discards released into the water during landing operations in the pier. At this location illegal catch is rare, and locals take care of the green turtles. Although this study did not explicitly examine the biotic and abiotic reasons explaining the residence times in a given habitat, these results highlight the importance of the use of neritic residences along the Southeast Pacific coast.

### Outlook and conservation issues

In this study, we emphasize the presence of green turtle aggregations at two neritic sites of the Peruvian coast. Unlike most of the studies in Peru on biological aspects of sea turtles whose source comes from interactions with fisheries [23], [30], [52]–[55], from poaching [29], [31], [34] or from stranding [56], we present results from a less disturbed situation. Nevertheless, some aspects still need research attention such as the degree of connectivity between sites and it should be assessed with further satellite tracking and genetic research. In addition, isotopic studies may reveal important aspects of the trophic ecology of this species such as the role of different upwelling regimes in food supply. Giving the high density of humans living in coastal areas, green turtles in neritic habitats will be more exposed to negative human interactions and although the green turtle is under the protection of international and Peruvian laws, there are threats at both sites. At El Ñuro the aggregation occurs very close to an artisanal fishing pier, so green turtles are constantly at risk of interacting with human debris and the potential for vessel collision is high. Furthermore, snorkeling with green turtles has become a regular tourist activity that may have undesirable consequences on the feeding and migratory behavior of this species. In Paracas, a traditional sea turtle fishery existed [29], and although now illegal, it still occurs in significant numbers (Javier Quiñones, unpublished data). Collision with vessels is a constant threat to green turtles in this site due to high amount of recreational boats (particularly Jet Ski) navigating the bay. The biological traits examined in our study (e.g., growth rate, body condition) suggest that green turtles inhabiting the coast of Peru thrive under excellent habitat conditions which lately may cause a positive impact on the recovery of the whole population. However, if the aforementioned anthropogenic impacts are not mitigated the endangered situation of this species might remain. We strongly encourage enhancing conservation and educational programs for this species especially within artisanal fishermen communities.

## Supporting Information

Table S1 Lengths, weight and growth rate and body condition index measurements of all individuals studied at el Ñuro.
(XLSX)
